# Bergapten Alleviates Periodontitis by Suppressing NLRP3 Inflammasome Activation via Mitophagy-Induced Disruption of NEK7-NLRP3 Interaction

**DOI:** 10.3390/antiox15101304

**Published:** 2026-10-09

**Authors:** Xiangbin Jia, Yuzhao Wang, Jiao Cao, Lifei Pan, Xiaoxuan Cui, Hanxue Shan, Yiqing Wang, Xiao Cao, Qin Zhou

**Affiliations:** 1 Key Laboratory of Shaanxi Province for Craniofacial Precision Medicine Research, College of Stomatology, Xi’an Jiaotong University, Xi’an 710004, China; jxb1946328@stu.xjtu.edu.cn (X.J.);; 2 Clinical Research Center of Shaanxi Province for Dental and Maxillofacial Diseases, College of Stomatology, Xi’an Jiaotong University, Xi’an 710004, China; 3 Department of Urology, The First Affiliated Hospital of Xi’an Jiaotong University, Xi’an 710061, China; 4 Key Laboratory for Tumor Precision Medicine of Shaanxi Province, The First Affiliated Hospital of Xi’an Jiaotong University, Xi’an 710061, China; 5 Engineering Research Center of Tibetan Medicine Detection Technology, Ministry of Education, School of Medicine, Xizang Minzu University, Xianyang 712082, China; 6 Department of Implant Dentistry, College of Stomatology, Xi’an Jiaotong University, Xi’an 710004, China

**Keywords:** periodontitis, bergapten, NLRP3 inflammasome, NEK7, mitophagy, mtROS

## Abstract

Periodontitis is a prevalent chronic inflammatory disease characterized by gingival inflammation and progressive alveolar bone loss. The NLRP3 inflammasome functions as a molecular mediator that drives its pathogenesis. Bergapten (BeG), a natural furanocoumarin, exhibits anti-inflammatory properties and bone-protective effects. This study demonstrates that BeG alleviates experimental periodontitis in mice by inhibiting the NLRP3 inflammasome. Mechanistically, BeG does not affect NLRP3 priming, dispersed trans-Golgi network (dTGN) localization, or oligomerization; instead, it induces mitophagy by inhibiting the AKT–mTOR axis and consequently suppresses the mtROS-dependent NEK7–NLRP3 interaction. Notably, BeG also suppressed cytosolic release of oxidized mitochondrial DNA (ox-mtDNA), a known endogenous ligand for NLRP3. This effect was attributed to BeG-mediated inhibition of NLRP3 activation, likely via blockade of GSDMD-NT mitochondrial localization. These findings deepen our understanding of the anti-inflammatory mechanisms of BeG and position it as a promising candidate for periodontitis treatment.

## 1. Introduction

Periodontitis is a chronic inflammatory oral disease characterized by the destruction of gingival tissue, the periodontal ligament, and alveolar bone, ultimately leading to tooth loss. Current treatment strategies for periodontitis primarily rely on mechanical therapy, which has limited efficacy and is often associated with disease recurrence [1]. Therefore, it is urgently necessary to identify effective therapeutic targets for periodontitis and develop novel drugs or therapies to inhibit its progression [2,3].

The NLRP3 inflammasome is a central mediator of periodontitis pathogenesis [4,5,6]. It is an intracellular multiprotein complex composed of the NLRP3 protein, NIMA-related kinase 7 (NEK7), apoptosis-associated speck-like protein containing a CARD (ASC) and pro-caspase-1. Its activation involves two steps: priming and activation. During priming, LPS induces transcriptional upregulation of NLRP3 and pro-IL-1β. Upon stimulation by secondary signals such as ATP, nigericin, or R837, NLRP3 is recruited to the dispersed trans-Golgi network (dTGN) where it oligomerizes into a closed cage-like structure. Subsequently, NEK7 binding induces a conformational change in NLRP3, exposing its PYD domain [7,8,9,10]. This enables the recruitment of ASC oligomers and the subsequent cleavage of caspase-1 into its active p20 and p10 subunits. These active caspase-1 fragments convert pro-IL-1β to mature IL-1β and process gasdermin D into its N-terminal fragment (GSDMD-NT), which oligomerizes to form plasma membrane pores, triggering pyroptosis and releasing mature IL-1β to drive inflammatory responses [11,12].

Mitochondrial dysfunction, potassium efflux, and lysosomal rupture are recognized as upstream events in NLRP3 activation. Although no consensus model fully explains NLRP3 inflammasome activation, most known NLRP3 activators disrupt mitochondrial redox homeostasis—resulting in excessive mtROS production and cytosolic release of ox-mtDNA, both established endogenous triggers of NLRP3 inflammasome assembly [12,13,14,15]. Moreover, antioxidant treatment consistently suppresses NLRP3 activation [16,17,18,19]. Collectively, these findings identify mitochondrial redox dysregulation as a critical upstream event initiating NLRP3 inflammasome assembly. Mitophagy is a selective process that removes dysfunctional mitochondria. It involves the recruitment of LC3-II to form a double-membrane autophagosome, which engulfs the damaged mitochondria and delivers them to lysosomes for degradation. Therefore, enhancing mitophagy has emerged as a promising therapeutic strategy to suppress NLRP3 inflammasome activation [20,21].

Bergapten (BeG) is a natural furanocoumarin that has gradually attracted increasing attention due to its broad pharmacological activities, including anti-inflammatory and bone protective effects [22]. A recent study reported that BeG inhibits NLRP3 activation and alleviates Escherichia coli-induced sepsis [23]. However, the molecular mechanism underlying BeG’s pharmacological effects remains incompletely defined, and its therapeutic effect on periodontitis has not yet been confirmed. This study reveals that BeG inhibits NLRP3 activation by disrupting the mtROS-dependent NEK7-NLRP3 interaction, likely through mitophagy induction via AKT-mTOR inhibition. Moreover, BeG exhibits therapeutic efficacy against experimental periodontitis in mice, highlighting its potential as a promising therapeutic candidate for periodontitis.

## 2. Materials and Methods

### 2.1. Reagents

LPS (cat. no. L2880) and ATP (cat. no. L2383) were purchased from Sigma-Aldrich (St. Louis, MO, USA). BeG (cat. no. HY-N0370), MHY1485 (cat. no. HY-B0795), MCC950 (cat. no. HY-12815), R837 (cat. no. HY-B0180A), chloroquine (cat. no. HY-17589A) and nigericin (cat. no. HY-127019) were purchased from MedChemExpress (Monmouth Junction, NJ, USA). M-CSF (cat. no. CB34) was purchased from Novoprotein (Suzhou, China). The antibodies used in the experiment are listed in Table S1.

### 2.2. Clinical Samples

The diagnosis of periodontitis was established according to the periodontal classification from the 2017 World Workshop [24]. Healthy periodontal tissues (*n* = 15) were harvested from systemically healthy volunteers undergoing elective extraction of permanent teeth for orthodontic reasons; all donors exhibited a full-mouth plaque index ≤ 25% and a full-mouth bleeding on probing (BOP) score ≤ 10%. Periodontal tissue (*n* = 15) was collected from patients with periodontitis who required tooth extraction due to non-retainable affected teeth. Demographic and clinical characteristics are shown in Table S3.

### 2.3. Animal Experiments

C57BL/6J mice (male, 8 weeks of age) were purchased from and bred at the Model Animal Research Center of XXX under specific pathogen-free housing conditions. Mice were randomly assigned to experimental groups (*n* = 5 per group) and acclimated for one week prior to the experiment. Experimental periodontitis was induced by oral ligature method. Briefly, mice were anesthetized with isoflurane (2%, 800 mL/min O_2_), and ligatures were placed around the maxillary second molars using 5-0 silk sutures. BeG was suspended in 0.5% sodium carboxymethyl cellulose (CMC-Na) and administered daily by oral gavage at doses of 40 and 80 mg/kg. Mice were euthanized 10 days after ligature placement.

### 2.4. Micro-CT Analysis

Maxillary samples were carefully dissected and fixed in 4% paraformaldehyde (PFA) for 48 h, followed by micro-computed tomography (micro-CT) analysis (nanoVoxel-1000, SANYING, Tianjin, China). The distance from the cementoenamel junction (CEJ) to the alveolar bone crest (ABC) at the second molar in each sample was measured to assess alveolar bone loss. All measurements were carried out in duplicate by a researcher blinded to the treatment groups, and the average of the two measurements was used for analysis.

### 2.5. Immunohistochemistry Staining

Mouse maxillary samples were processed for paraffin embedding and sectioning. Immunohistochemistry (IHC) was performed using SABC-POD kit (SA1028, Boster, Wuhan, China). Briefly, sections were incubated with enzyme complexes at 37 °C for 30 min and treated with 3% hydrogen peroxide (H_2_O_2_) for 30 min. After blocking, the sections were incubated with primary antibodies overnight at 4 °C, then incubated with secondary antibody at room temperature for 1 h. Positive signals were visualized using a DAB staining kit (Boster, AR1027). Images were captured by a slide scanning system (VS200, OLYMPUS, Tokyo, Japan). The percentage of positive area in IHC was measured by a researcher blinded to the treatment group using ImageJ v1.54p software (U.S. National Institutes of Health, Bethesda, MD, USA).

### 2.6. Cell Culture and Stimulation

All cells were cultured in an incubator containing 5% CO_2_ at 37 °C. Bone marrow was isolated from adult mice and cultured in high-glucose Dulbecco’s modified Eagle medium (DMEM) supplemented with 10% fetal bovine serum (FBS), 20 ng/mL M-CSF, and 1% penicillin-streptomycin for 5 days to induce differentiation into bone marrow–derived macrophages (BMDMs). The HeLa, HEK293T, and J774A.1 cell lines were obtained from Procell Life Science & Technology Co., Ltd. (Wuhan, China) and maintained in high-glucose DMEM supplemented with 10% FBS.

For NLRP3 inflammasome activation, macrophages were primed with LPS (200 ng/mL for BMDMs, 500 ng/mL for J774A.1) for 4 h, followed by exposure to ATP (4 mM) or R837 (200 μM) for 1 h. BeG (5–20 μM), MHY1485 (2 μM) were added 2 h or 4 h prior to LPS priming, and MCC950 (100 nM) was added 0.5 h prior to ATP treatment. Supernatants and cell lysates were collected and analyzed for NLRP3 activation and IL-1β secretion by immunoblotting.

### 2.7. Targeting of NEK7 by CRISPR-Cas9-Mediated Genome Editing

To generate a NEK7 knockout cell line, lentiCRISPRv2 stocks containing the target sequence 5′-ATAGAAAAGAAAATTGGTCG-3′ were used to transduce J774A.1 cells. Single clones of blasticidin -resistant cells were identified by immunoblotting.

### 2.8. Plasmids and Transfection

cDNAs encoding mouse Nek7, Nlrp3, and Pycard were amplified by PCR from a murine cDNA library and subcloned into the pcDNA3.1 vector with His, GFP, Myc or HA tags, respectively. HEK293T cells were seeded into 6-well plates in complete culture medium and incubated overnight prior to transfection. Thereafter, cells were transfected with NLRP3-GFP (800 ng/well), His-NLRP3 (800 ng/well), NEK7-Myc (800 ng/well), and ASC-HA (40 ng/well) using jetPRIME transfection reagent (101000046, Polyplus, Illkirch, France). After 36 h, cells were treated with BeG (20 μM) for 6 h and then lysed with NP-40 lysis buffer.

### 2.9. Western Blot and Co-Immunoprecipitation

An equal volume of medium supernatants from treated macrophages was precipitated using trichloroacetic acid (TCA). Cells were harvested in RIPA buffer (P0013B, Beyotime, Shanghai, China), and lysates were resolved by SDS-PAGE, transferred to PVDF membranes. After overnight incubation with primary antibodies, the membranes were incubated with HRP-conjugated secondary antibodies for 1 h. Protein expression was assessed using an automatic chemiluminescence imaging system (Bio-Rad, ChemiDoc) (ChemiDoc, Bio-Rad, Hercules, CA, USA). For co-immunoprecipitation, cells were lysed in NP-40 lysis buffer for 30 min on ice. The lysates were then centrifuged at 13,000× *g* for 15 min at 4 °C. The supernatant was collected and mixed with primary antibodies overnight at 4 °C. Subsequently, the samples were incubated with Protein A/G agarose beads (P2055, Beyotime, Shanghai, China) for 1 h at 4 °C. After washing, the immunoprecipitated complexes were eluted using SDS loading buffer at 95 °C for 5 min, and subsequently analyzed by Western blotting. Western blot assays were conducted in three independent replicates, and representative images are presented in the figures.

### 2.10. Semi-Denaturing Detergent Agarose Gel Electrophoresis (SDD-AGE)

The oligomerization of NLRP3 was analyzed according to the published protocol [25]. Cells were lysed with Triton X-100 lysis buffer (0.5% Triton X-100, 50 mM Tris–HCl, 150 mM NaCl, 10% glycerol, 1 mM PMSF, and protease inhibitor cocktail). The lysates were then resuspended in 1× sample buffer (0.5× TBE, 10% glycerol, 2% SDS, and 0.0025% bromophenol blue) and loaded onto a vertical 1.5% agarose gel. After electrophoresis in running buffer (1× TBE and 0.1% SDS) for 1 h at a constant voltage of 80 V and 4 °C, proteins were transferred to PVDF membranes for immunoblotting. 1× TBE buffer contains 89 mM Tris (pH 8.3), 89 mM boric acid, and 2 mM EDTA.

### 2.11. Immunofluorescence Staining

Cells were washed and fixed with 4% paraformaldehyde for 15 min and then permeabilized using Triton X-100 Buffer (P0096, Beyotime, Shanghai, China) for 10 min. Tissue sections were incubated with enzyme complexes at 37 °C for 30 min. After blocking with QuickBlock™ blocking buffer (P0260, Beyotime, Shanghai, China) for 15 min, cells and tissue sections were incubated with a Primary antibodies overnight at 4 °C, followed by staining with Alexa Fluor secondary fluorescent antibodies and DAPI. To assess inflammatory cell death, cells were stained with PI (C1008, Beyotime, Shanghai, China) and Hoechst 33342 (C1028, Beyotime, Shanghai, China) for 15 min. Images of cells were acquired by a confocal microscope (FV3000, Olympus, Tokyo, Japan).

### 2.12. Mitochondrial Function

Mitochondrial membrane potential was assessed by JC-1 staining (M8650, Solarbio, Beijing, China) according to the manufacturer’s instructions. mtROS were measured using MitoSOX (40778ES50, Yeasen, Shanghai, China), and cells were treated with 5 μM MitoSOX for 30 min. After three washes with PBS, the assay buffer was added, and the cells were observed using a fluorescence microscope.

### 2.13. Cellular Fractionation and Measurement of Cytosolic mtDNA

The cytosolic mtDNA was analyzed according to the published protocol [18]. The treated macrophages were collected and divided into two parts, 10% was used for extracting whole-cell DNA by Dneasy Blood & Tissue Kit (69504, QIAGEN, Hilden, Germany), and 90% was retained for extracting mitochondrial and cytosolic fractions. Briefly, cells resuspended in mitochondrial extraction buffer (220 mM mannitol, 70 mM sucrose, 20 mM HEPES-KOH, pH 7.5, 1 mM EDTA and 2 mg/mL BSA, 1× phosphatase inhibitor, 1× protease inhibitor cocktail) were passed through a 25-G syringe 20 times on ice. The homogenates were centrifuged at 1000× *g* for 10 min, the supernatant was then centrifuged at 10,000× *g* for 20 min to obtain the mitochondrial pellet and the cytosolic fraction in the supernatant. Cytosolic DNA was then isolated from the cytosolic fraction using Dneasy Blood & Tissue Kit. Quantitative PCR analysis of whole-cell and cytosolic DNA was performed using nuclear DNA primers (Tert) and mitochondrial DNA primers (D-loop) to measure the levels of cytoplasmic mtDNA. The primer sequences used in this study are listed in Table S2.

### 2.14. Detection of Autophagic Flux and Mitophagy

Autophagic flux was assessed following the published protocol [26]. HeLa cells were transiently transfected with the mCherry-EGFP-LC3 plasmid using jetPRIME transfection reagent. At 36 h post-transfection, cells were treated with BeG (20 μM, 6 h) alone or in combination with chloroquine (20 μM, 6 h). Subsequently, cells were fixed with 4% paraformaldehyde for 15 min at room temperature. Images of cells were acquired by a confocal microscope. In cells transfected with mCherry-EGFP-LC3, EGFP fluorescence is quenched in the acidic lysosomal lumen, whereas mCherry fluorescence remains stable. Consequently, autophagosomes display both mCherry and EGFP puncta, whereas autolysosomes exhibit mCherry-only puncta. Thus, an increase in mCherry-only puncta specifically indicates enhanced autophagic flux. Mitophagy in BMDM and J774A.1 cells was observed by immunofluorescence colocalization staining using antibodies against LC3 and the mitochondrial marker TOM20.

### 2.15. RNA Sequencing

After drug treatment, total RNA was extracted from BMDMs using TRIzol reagent. Three biological replicates were included for each group. Sequencing was performed on an Illumina NovaSeq 6000 platform through a commercial service provided by Genergy Biotechnology Co., Ltd. (Shanghai, China). After quality filtering, each sample yielded no fewer than 2 × 10^7^ clean reads. Differentially expressed transcripts were identified using the MA-plot-based method with random sampling (MARS) in the DEGseq package, with a significance threshold of adjusted *p* < 0.05 (padj) and an absolute fold change ≥ 1.5. Gene Ontology (GO) functional enrichment analysis was performed using the R package clusterProfiler (v4.8.1). For visualization, raw counts were corrected for gene length and sequencing depth to obtain transcripts per million (TPM) values, which were then Z-score normalized across samples for heatmap generation. Volcano plots and heatmaps were generated using R software (version 4.0.3). The original data presented in the study are openly available in the NCBI Sequence Read Archive (SRA) under BioProject accession number PRJNA1493853.

### 2.16. Statistical Analyses

Data were presented as the mean ± standard deviation (SD). Statistical analyses were conducted using GraphPad Prism v10.1.2 (GraphPad Software, Boston, MA, USA). Normality was assessed with the Shapiro–Wilk test, and homogeneity of variances was evaluated using Levene’s test. As all data satisfied these assumptions, differences among multiple groups were analyzed by one-way analysis of variance (ANOVA) followed by Tukey’s post hoc test. A *p* value less than 0.05 was considered statistically significant.

## 3. Results

### 3.1. Bergapten Suppresses NLRP3 Inflammasome Activation

**Figure 1:**
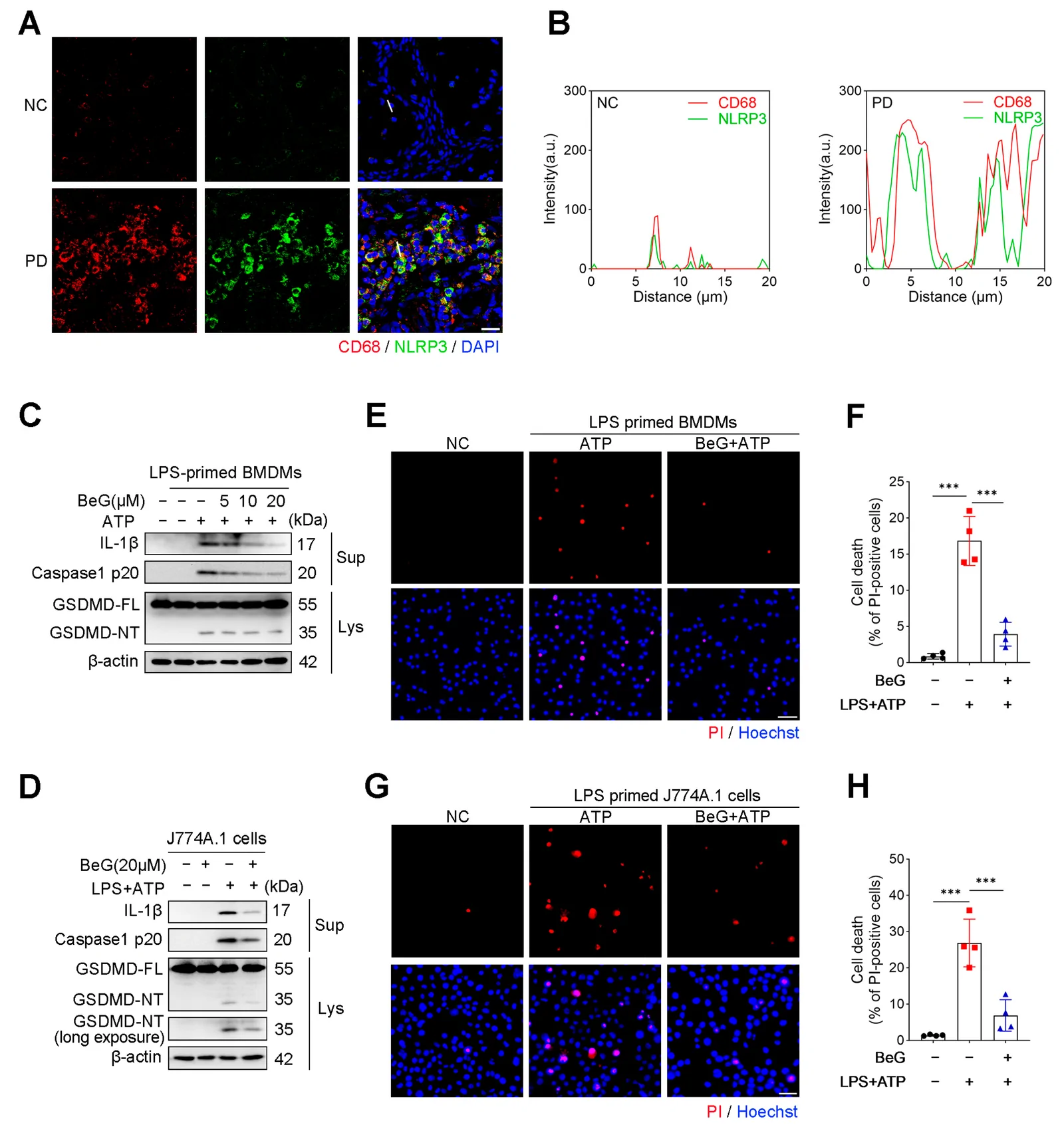
Bergapten suppresses NLRP3 inflammasome activation. (**A**,**B**) Representative immunofluorescence images revealed the co-localization of NLRP3 and CD68 in gingival tissues from healthy individuals and patients with periodontitis (*n* = 3). Fluorescence intensity profiles along the white lines were shown on the right. Scale bar, 20 µm. (**C**,**D**) Mature IL-1β and cleaved caspase-1 (p20) in cell-free supernatants, NLRP3 and GSDMD in lysates, were analyzed by Western blot in LPS/ATP-stimulated BMDMs and J774A.1 cells. (**E**–**H**) Representative immunofluorescence images of cell death in LPS/ATP-stimulated BMDMs and J774A.1 cells were obtained by staining with propidium iodide (PI). Each group used 12 images from four independent experiments to calculate the proportion of PI-positive cells. Scale bar, 40 μm. Mean values were used to calculate SD (error bars) and *p* values. Statistical significance was indicated as follows: *** *p* < 0.001.

**Figure 2:**
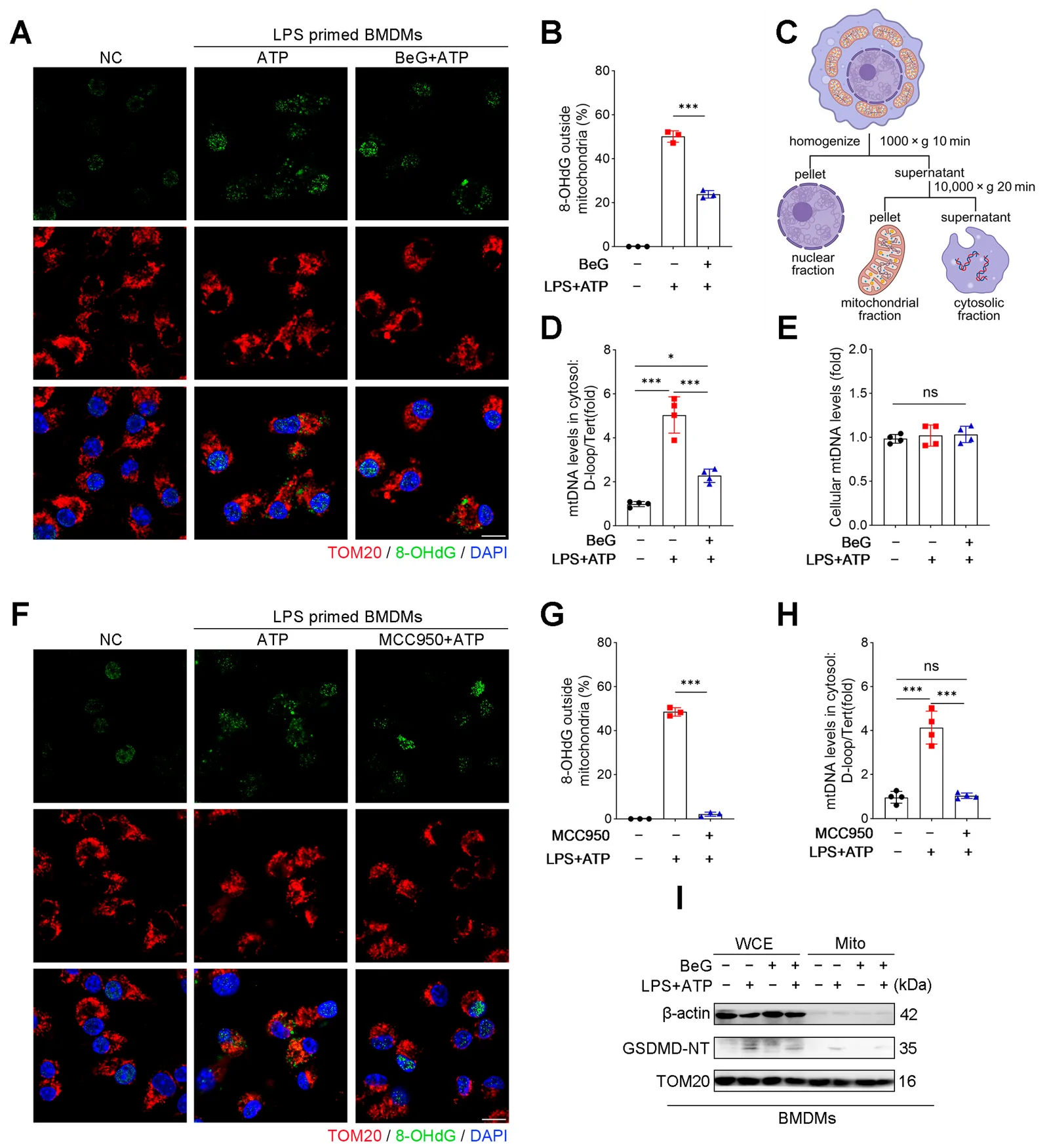
Bergapten inhibits GSDMD-NT-mediated mitochondrial pore formation, thereby attenuating cytosolic leakage of ox-mtDNA. (**A**,**B**) Representative immunofluorescence images of 8-OHdG and TOM20 staining in LPS/ATP-stimulated BMDMs with or without BeG treatment. Scale bar, 10 μm. The percentage of cells with 8-OHdG localized outside the mitochondria was quantified from 30 images across three independent experiments. (**C**) Schematic representation of cell lysis and centrifugation for subcellular fractionation. (**D**,**E**) DNA was extracted from whole-cell lysates and cytosolic fractions of BMDMs pretreated with BeG, and mtDNA levels in both the cytosol and total cellular compartments were quantified by qPCR. Statistical analysis was based on four independent experiments per group. (**F**,**G**) Representative immunofluorescence images of 8-OHdG and TOM20 staining in LPS/ATP-stimulated BMDMs with or without MCC950 treatment. (**H**) DNA was extracted from whole-cell lysates and cytosolic fractions of BMDMs pretreated with MCC950, and cytosolic mtDNA was quantified by qPCR. (**I**) Immunoblotting was used to detect the protein levels of GSDMD-NT, β-actin and TOM20 in whole cell extractions and mitochondrial fractions of BMDMs pretreated with BeG. Mean values were used to calculate SD (error bars) and *p* values. Statistical significance was indicated as follows: ns, not significant, * *p* < 0.05, *** *p* < 0.001.

Histological analysis of gingival tissues from periodontitis patients and healthy individuals revealed significantly upregulated NLRP3 expression in periodontitis tissues, predominantly localized to CD68-positive macrophages (Figure 1A,B). In macrophages, BeG-mediated inhibition of the NLRP3 inflammasome dose-dependently suppressed caspase-1 cleavage and IL-1β secretion (Figure S1A,B and Figure 2C,D). Consistent with this, BeG markedly inhibited pyroptosis, as evidenced by reduced PI staining and diminished GSDMD cleavage (Figure 2C–H). Collectively, these results demonstrate that BeG effectively suppresses NLRP3 inflammasome activation.

### 3.2. Bergapten Inhibits GSDMD-NT-Mediated Mitochondrial Pore Formation, Thereby Attenuating Cytosolic Leakage of Ox-mtDNA

Mitochondrial oxidative stress induces oxidative damage to mtDNA, generating 8-hydroxy-2′-deoxyguanosine (8-OHdG). Once released into the cytosol, ox-mtDNA binds to NLRP3 and promotes NLRP3 inflammasome activation [18,27]. We first assessed whether BeG modulates cytosolic release of ox-mtDNA. Immunofluorescence showed that BeG significantly reduced the proportion of BMDMs with ox-mtDNA leakage (Figure 2A,B). Subcellular fractionation combined with qPCR (Figure 2C–E) revealed that BeG did not affect total mtDNA levels but reduced cytoplasmic mtDNA after LPS/ATP stimulation, indicating that BeG inhibits ox-mtDNA leakage into the cytosol. Since GSDMD-NT also forms pores in the mitochondrial membrane to release mtDNA [28], it remains unclear whether BeG-mediated inhibition of ox-mtDNA leakage is a cause or consequence of NLRP3 blockade.

Moreover, the specific NLRP3 inhibitor MCC950 completely blocked the release of ox-mtDNA (Figure 2F–H), indicating that this process strictly depends on NLRP3 activation. Consistently, BeG treatment significantly reduced mitochondrial localization of GSDMD-NT (Figure 2I). BeG may prevent cytosolic release of ox-mtDNA by inhibiting NLRP3 activation, likely by reducing GSDMD-NT cleavage and subsequent mitochondrial damage. However, the upstream mechanism by which BeG suppresses NLRP3 activation remains to be elucidated.

### 3.3. Bergapten Disrupts the NEK7–NLRP3 Interaction and Thereby Inhibits Downstream ASC Recruitment

**Figure 3:**
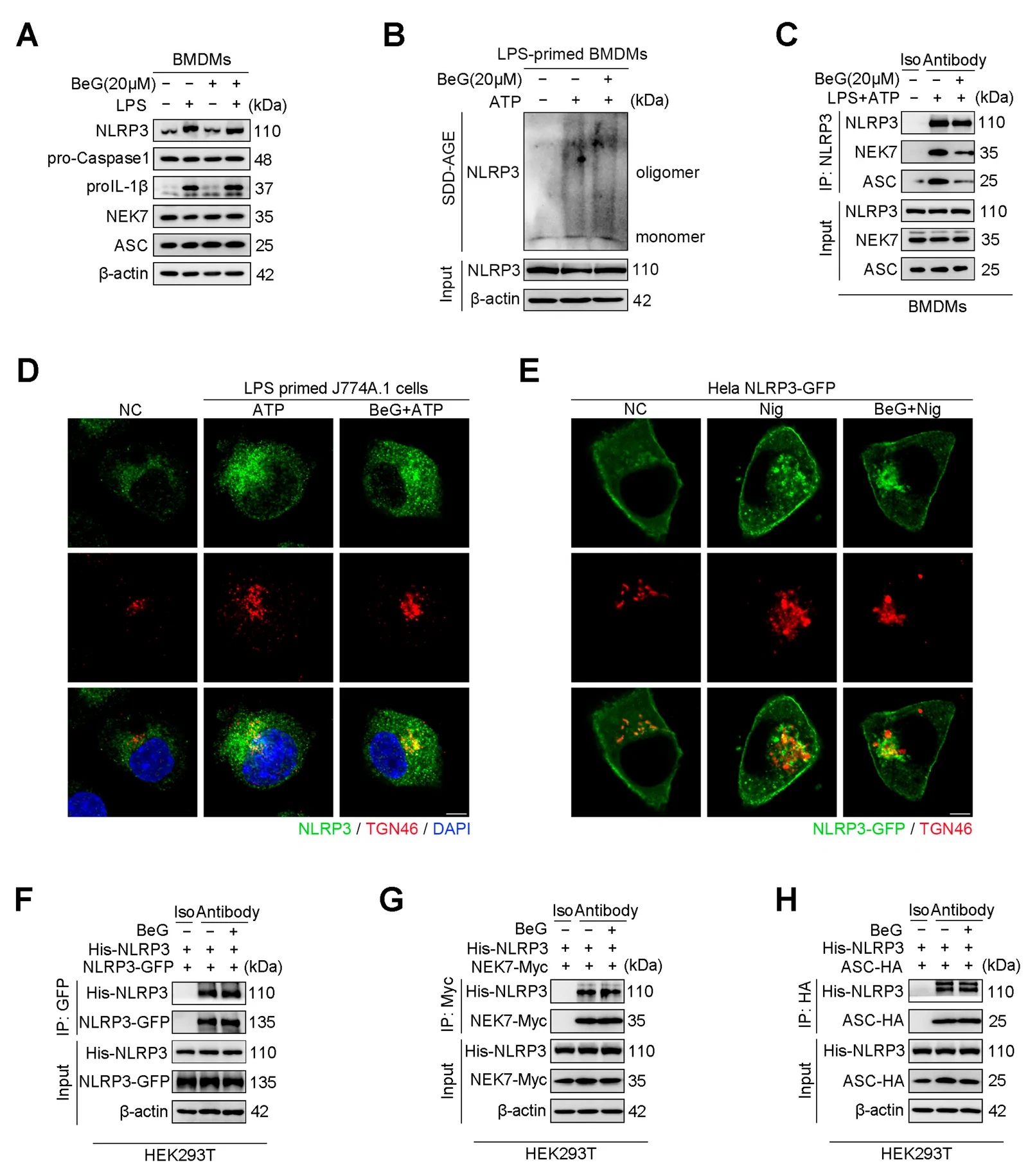
Bergapten disrupts the NEK7–NLRP3 interaction and thereby inhibits downstream ASC recruitment. (**A**) Western blot analysis of whole-cell lysates from BMDMs pretreated with BeG and primed with LPS (200 ng/mL, 4 h) quantified protein levels of NLRP3, pro-caspase-1, NEK7, ASC, and pro-IL-1β. (**B**) SDD-AGE was used to assess the effect of BeG on NLRP3 oligomerization in LPS/ATP- stimulated BMDMs. (**C**) Co-immunoprecipitation and Western blotting assessed BeG’s effect on the NEK7–NLRP3 and NLRP3–ASC interactions in LPS/ATP- stimulated BMDMs. (**D**,**E**) Immunofluorescence analysis of the effect of BeG on NLRP3 recruitment to the dTGN in LPS/ATP-stimulated J774A.1 cells (**D**) and in HeLa cells stably expressing NLRP3-GFP following stimulation with nigericin (10 μM, 1 h) (**E**). Scale bar, 5 μm. (**F**–**H**) Co-immunoprecipitation assessed the effect of BeG on the interaction between His-NLRP3 and NLRP3-GFP (**F**), NEK7-Myc (**G**), or ASC-HA (**H**) in HEK293T cells.

To elucidate the mechanism underlying BeG-mediated suppression of NLRP3 inflammasome activation, we performed a systematic investigation. First, Western blot analysis revealed that BeG pretreatment did not alter the protein levels of NLRP3, pro-IL-1β, NEK7, ASC, or pro-caspase-1 in LPS-stimulated BMDMs (Figure 3A), indicating that BeG affects neither inflammasome priming nor the basal expression of its core components. Next, we assessed BeG’s effect on the inflammasome activation phase and found that it neither impaired NLRP3 recruitment to the dTGN (Figure 3D,E) nor disrupted NLRP3 oligomerization (Figure 3B), but attenuated the NEK7–NLRP3 and NLRP3–ASC interactions (Figure 3C).

To determine whether BeG competitively inhibits the interaction between NEK7, NLRP3, and ASC, we transfected plasmids encoding NEK7, NLRP3, and ASC into HEK293T cells, in which these proteins spontaneously assemble into functionally active complexes [29]. However, BeG did not disrupt the spontaneous interaction among NLRP3 inflammasome components in HEK293T cells, thereby excluding competitive inhibition as a mechanism of action (Figure 3F–H). Since NLRP3 conformational opening and PYD domain exposure depend on NEK7 interaction, which is required for subsequent ASC recruitment [9], the observed decrease in NLRP3–ASC interaction is primarily caused by BeG-mediated inhibition of the NEK7–NLRP3 interaction. Collectively, BeG may indirectly inhibit NLRP3 inflammasome activation by modulating upstream signaling events essential for NEK7–NLRP3 interaction.

### 3.4. Bergapten Disrupts the NEK7-NLRP3 Interaction by Suppressing mtROS Overproduction

**Figure 4:**
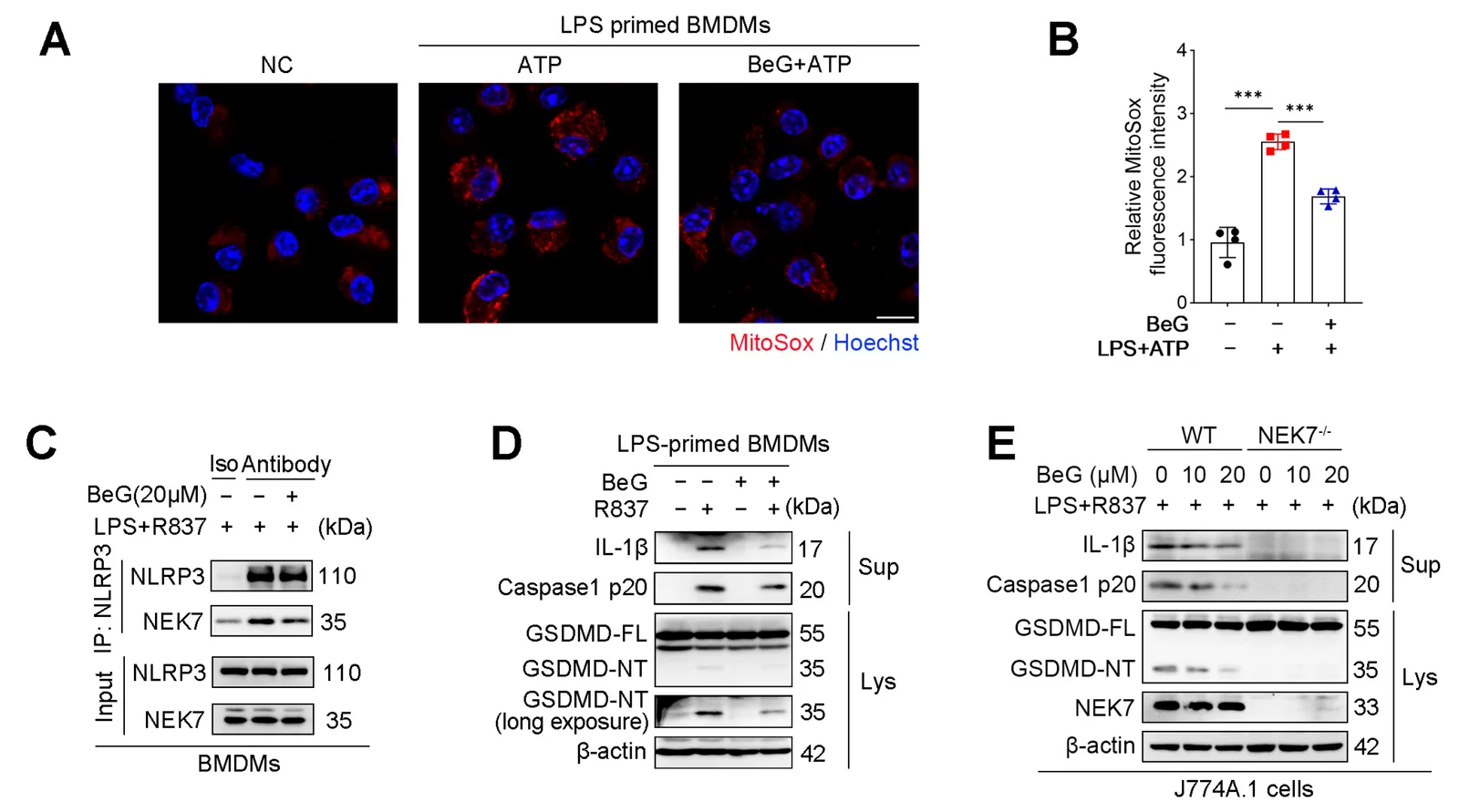
Bergapten disrupts the NEK7-NLRP3 interaction by suppressing mtROS overproduction. (**A**,**B**) Representative images of MitoSOX staining and quantitative analysis of average fluorescence intensity in BMDMs. Scale bar, 10 μm. Each group used 20 images from four independent experiments for statistical analysis. Mean values were used to calculate SD (error bars) and *p* values. Statistical significance was indicated as follows: ns, not significant, *** *p* < 0.001. (**C**) Co-immunoprecipitation and Western blotting assessed BeG’s effect on the NEK7–NLRP3 interaction in LPS/R837- stimulated BMDMs. (**D**) Mature IL-1β and cleaved caspase-1 (p20) in cell-free supernatants, GSDMD in lysates, were analyzed by immunoblotting in LPS/R837-stimulated BMDMs. (**E**) Mature IL-1β and cleaved caspase-1 (p20) and GSDMD were analyzed by immunoblotting in LPS/R837-stimulated wild-type (WT) or Nek7−/− J774A.1 cells.

Given that mtROS serve as an upstream signal that governs the NEK7–NLRP3 interaction [30], we measured mtROS levels using MitoSOX Red. The results showed that BeG significantly suppressed mtROS production induced by LPS and ATP stimulation (Figure 4A,B). Since ATP activates NLRP3 through both mtROS generation and potassium efflux, we used R837, an agonist that specifically triggers NLRP3 activation by inhibiting mitochondrial complex I and inducing mtROS [31], thereby excluding the interference of potassium efflux. Results demonstrate that BeG suppresses R837-induced NEK7–NLRP3 interaction, GSDMD cleavage, and IL-1β secretion (Figure 4C,D). In Nek7−/− J774A.1 cells, the dose-dependent inhibition of LPS/R837-induced NLRP3 activation by BeG was abolished (Figure 4E), indicating that NEK7 is an essential upstream mediator through which BeG suppresses mtROS production and subsequent NLRP3 activation. Collectively, BeG suppresses the NEK7–NLRP3 interaction by inhibiting mtROS generation.

### 3.5. Bergapten Induces Mitophagy and Suppresses Excessive mtROS Production by Inhibiting the AKT-mTOR Signaling Pathway

**Figure 5:**
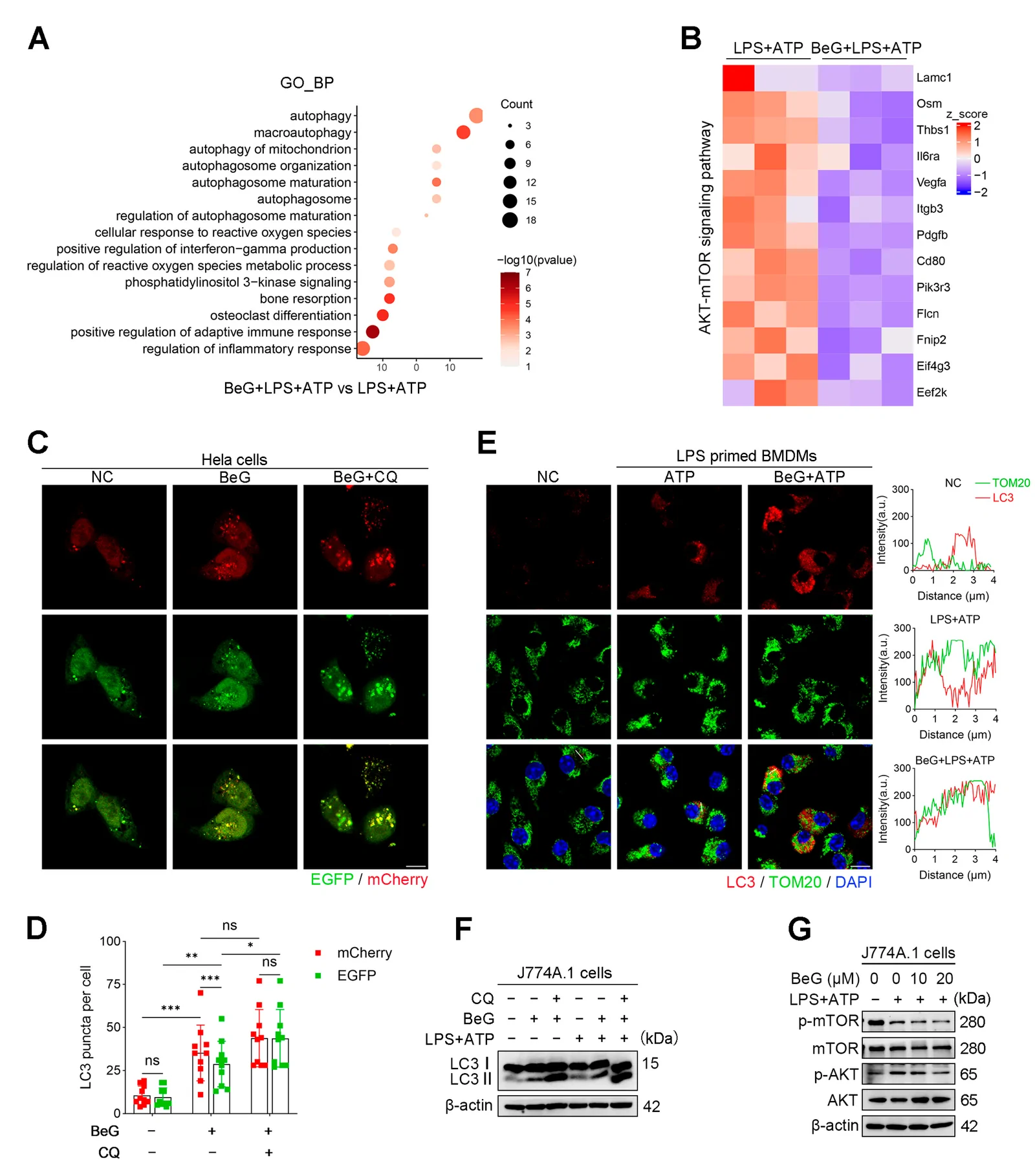
Bergapten induces mitophagy and suppresses excessive mtROS production by inhibiting the AKT-mTOR signaling pathway. (**A**) GO pathway enrichment analysis (biological process, BP) was performed using differentially expressed genes (DEGs) from LPS/ATP-stimulated BMDMs with or without BeG pretreatment. (**B**) A heatmap presents the relative mRNA expression of genes associated with AKT–mTOR signaling pathway. (**C**,**D**) HeLa cells transfected with the mCherry EGFP LC3 reporter plasmid were treated with BeG (20 μM) or lysosomal inhibitor chloroquine (CQ; 10 μM) for 6 h. Autophagic flux was then analyzed by confocal microscopy. The numbers of mCherry and EGFP positive puncta in representative images were quantified. Scale bar, 10 μm. Each group used 30 cells from three independent experiments for statistical analysis. Mean values were used to calculate SD (error bars) and *p* values. Statistical significance was indicated as follows: * *p* < 0.05, ** *p* < 0.01, *** *p* < 0.001. (**E**) Representative immunofluorescence images of LC3 and TOM20 staining in BMDMs. Scale bar, 10 μm. Fluorescence intensity profiles along the indicated white lines are shown on the right. (**F**) Immunoblotting analysis of LC3 in J774A.1 cells treated as indicated in the image, chloroquine (CQ) was used as a positive control. (**G**) Immunoblotting analysis of phosphorylated AKT (*p*-AKT) and phosphorylated mTOR (*p*-mTOR) levels in J774A.1 cells pretreated with different doses of BeG prior to LPS/ATP stimulation.

Given that BeG exhibits negligible intrinsic capacity to scavenge free radicals or ROS [32], we performed transcriptome sequencing to elucidate the mechanism through which BeG suppresses mtROS production in LPS/ATP-stimulated BMDMs. GO analysis revealed that differentially expressed genes in the BeG-treated group were significantly enriched in biological processes related to autophagy and mitophagy (Figure 5A). Heatmap analysis also showed that BeG significantly upregulated the mRNA expression of key regulatory genes for autophagosome maturation and mitophagy (Figure S2A,B). Furthermore, BeG significantly increased the numbers of both mCherry-EGFP-positive puncta (autophagosomes) and mCherry-only puncta (autolysosomes) (Figure 5C,D) in HeLa cells expressing the mCherry-EGFP-LC3 reporter, and promoted the conversion of LC3-I to LC3-II under both basal and inflammatory conditions in J774A.1 cells (Figure 5F). These findings demonstrate that BeG significantly enhances autophagic flux. Autophagosomes can also sequester the NLRP3 inflammasome and mediate its lysosomal degradation [21]. However, BeG did not induce significant co-localization between LC3-positive autophagosomes and NLRP3 specks (Figure S4A). Moreover, BeG increased the colocalization of mitochondria with LC3 in LPS/ATP-stimulated BMDMs and J774A.1 cells (Figure 5E and Figure S3A) and alleviated the reduction in mitochondrial membrane potential (Figure S3B). Therefore, BeG-induced autophagy preferentially clears damaged mitochondria rather than the NLRP3 inflammasome.

mTOR negatively regulates autophagy and mitophagy, with its phosphorylation directly controlled by AKT or AMPK phosphorylation [33,34]. Results demonstrate that BeG suppresses the expression of genes in the AKT-mTOR pathway and inhibited the phosphorylation of AKT and mTOR (Figure 5B,G). Moreover, BeG did not enhance AMPK phosphorylation—the canonical negative regulator of mTOR signaling (Figure S4B,C). Collectively, BeG promotes mitophagy by inhibiting the AKT-mTOR pathway, thereby eliminating damaged mitochondria and preventing excessive production of mtROS.

### 3.6. mTOR Activator MHY1485 Reverses Both Bergapten-Induced Mitophagy and NLRP3 Inhibition

**Figure 6:**
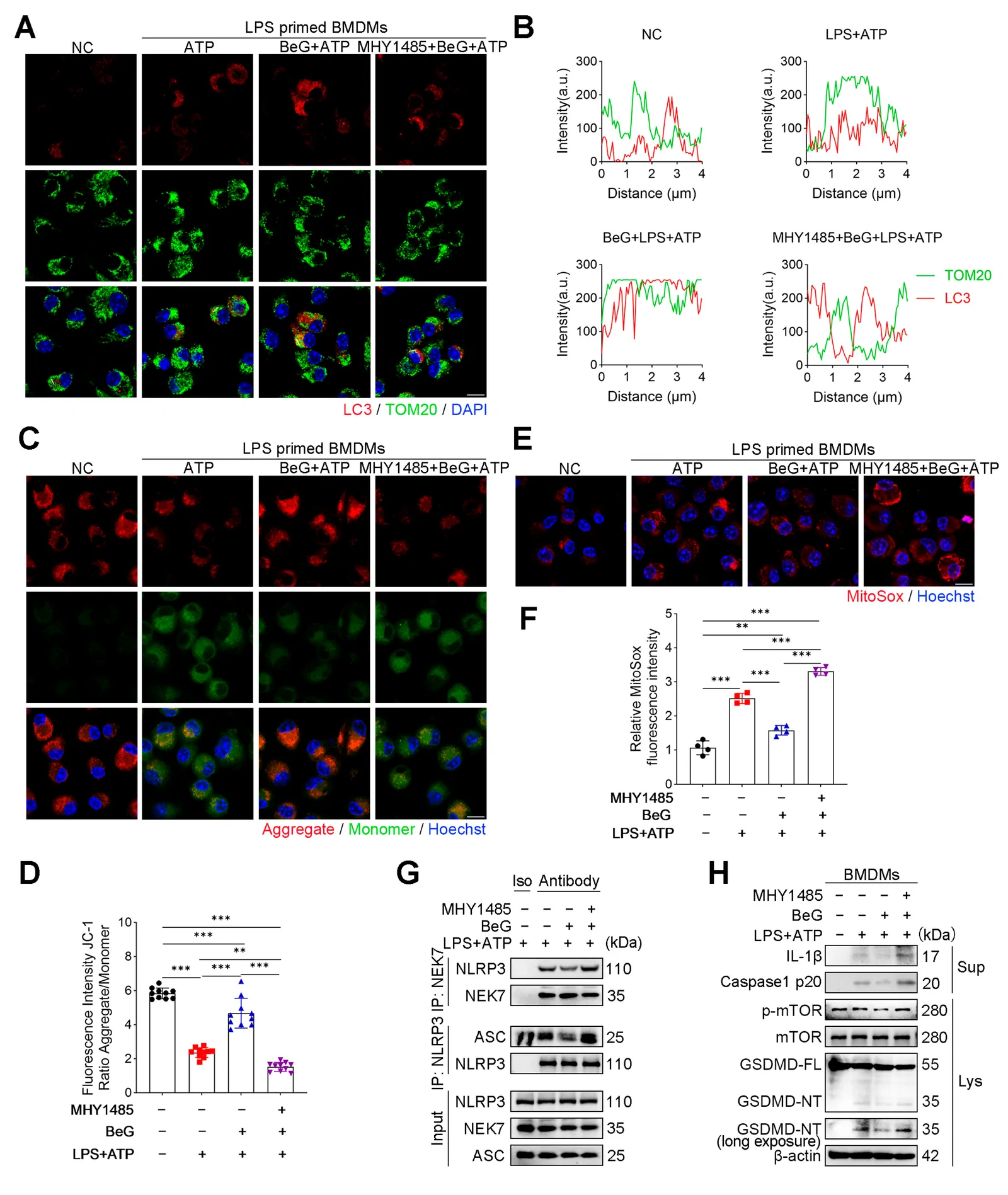
mTOR activator MHY1485 reverses both bergapten-induced mitophagy and NLRP3 inhibition. (**A**,**B**) Representative immunofluorescence images of LC3 and TOM20 staining in BMDMs pretreated with BeG and MHY1485. Fluorescence intensity profiles along the white lines are shown on the right. Scale bar, 10 μm. (**C**,**D**) Mitochondrial membrane potential in BMDMs was assessed using JC-1 staining. Scale bar, 10 μm. Each group used 10 images from three independent experiments for statistical analysis. Mean values were used to calculate SD (error bars) and *p* values. Statistical significance was in-dicated as follows: ** *p* < 0.01, *** *p* < 0.001. (**E**,**F**) Representative MitoSOX staining images and quantitative analysis of mean fluorescence intensity in BMDMs. Scale bar, 10 μm. Each group used 20 images from four independent experiments for statistical analysis. (**G**) Co-immunoprecipitation was used to assess NEK7–NLRP3 and NLRP3–ASC interactions in BMDMs. (**H**) Mature IL-1β and cleaved caspase-1 (p20) were detected by immunoblotting in supernatants of BMDMs, while *p*-mTOR and GSDMD were analyzed in whole-cell lysates.

To further clarify the role of mTOR in BeG-mediated NLRP3 inflammasome inhibition, we used the mTOR agonist MHY1485 for intervention. In LPS/ATP-stimulated BMDMs, MHY1485 blocked BeG-induced mitochondrial-LC3 colocalization (Figure 6A,B) and aggravated mitochondrial membrane potential loss (Figure 6C,D), indicating that MHY1485 blocks BeG-induced mitophagy. Moreover, MHY1485 reversed BeG-mediated suppression of LPS/ATP-induced mtROS production (Figure 6E,F), NEK7–NLRP3 and NLRP3-ASC interactions (Figure 6G), GSDMD cleavage, and IL-1β secretion (Figure 6H). Collectively, mTOR is a critical mediator of BeG’s effects on promoting mitophagy and suppressing NLRP3 activation.

### 3.7. Bergapten Alleviates Ligature-Induced Periodontitis

**Figure 7:**
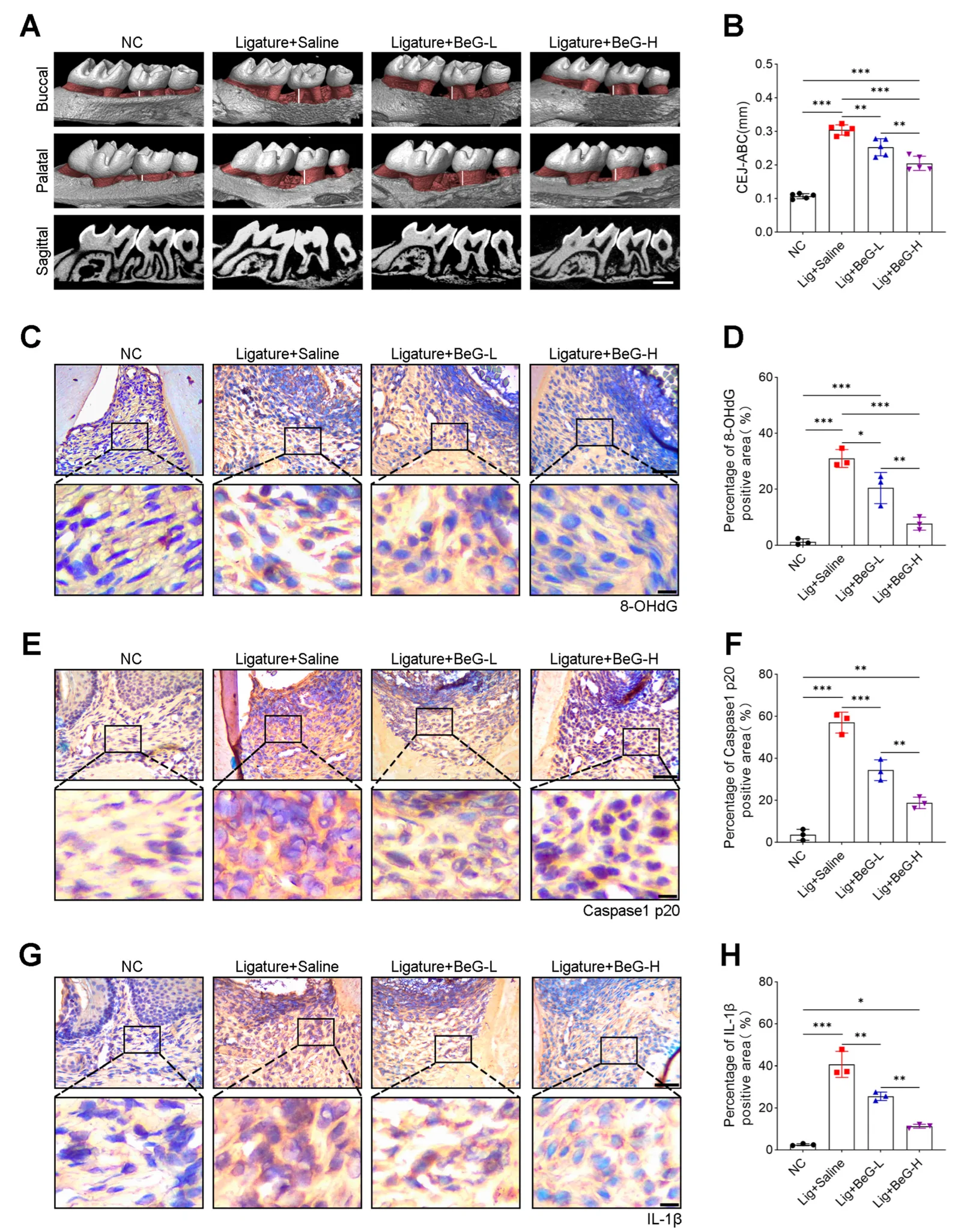
Bergapten alleviates ligature-induced periodontitis. (**A**,**B**) Micro-CT reconstruction of maxillary alveolar bone is shown, with red areas highlighting the range of CEJ-ABC. Scale bar, 500 μm. Five mice per group were used for statistical analysis. (**C**–**H**) Representative IHC images and quantification of 8-OHdG (**C**,**D**), cleaved caspase-1 (p20) (**E**,**F**), and IL-1β (**G**,**H**) expression in the gingival tissues of mice (*n* = 3). Scale bars: 50 μm (upper), 10 μm (below). IHC results from tissue sections of three mice per group were statistically analyzed. Values are presented as mean ± SD. Statistical significance was indicated as follows: * *p* < 0.05, ** *p* < 0.01, *** *p* < 0.001.

To evaluate the in vivo therapeutic potential of BeG, periodontitis was induced in C57BL/6 mice via ligature placement, and mice were subsequently treated with BeG at two doses: 40 mg/kg (low) and 80 mg/kg (high). Micro-CT revealed that ligature-induced alveolar bone loss was attenuated by BeG, indicating protection against periodontal bone resorption (Figure 7A,B). Furthermore, ligature-induced periodontitis significantly increased the levels of 8-OHdG, cleaved caspase-1 (p20), and IL-1β in periodontal tissues, suggesting enhanced oxidative stress and NLRP3 inflammasome activation, and these pathological elevations were effectively suppressed by BeG treatment (Figure 7C–H). Therefore, BeG effectively alleviated periodontal inflammation and alveolar bone loss by inhibiting NLRP3 inflammasome activation.

## 4. Discussion

Pharmacological targeting of the NLRP3 inflammasome represents a promising therapeutic strategy for periodontitis. BeG, a naturally occurring coumarin abundant in citrus fruits, has recently been shown to inhibit NLRP3 activation. Therefore, elucidating its precise molecular mechanism is essential to realizing its translational potential.

Previous studies have established ox-mtDNA as a key endogenous ligand required for NLRP3 activation [18,35,36,37]. However, our data show that cytosolic ox-mtDNA release is entirely dependent on NLRP3 activation, at least in its early phase. Considering that ox-mtDNA can bind and promote NLRP3 activation, this study regards ox-mtDNA as a non-essential activator of NLRP3. By inhibiting NLRP3 activation and reducing the mitochondrial localization of GSDMD-NT, BeG disrupts the ox-mtDNA-mediated feedback loop that amplifies NLRP3 activation.

BeG has been reported to inhibit NF-κB signaling [38,39], a canonical pathway for NLRP3 inflammasome priming. However, our results show that BeG fails to suppress LPS-induced expression of NLRP3, pro-IL-1β in BMDMs, possibly due to differences in cell type or cell model. Meanwhile, BeG does not impair NLRP3 translocation to the dTGN or its subsequent oligomerization.

The central mechanistic finding of this study is that BeG inhibits the assembly and activation of the NLRP3 inflammasome by disrupting the NEK7-NLRP3 interaction. Notably, BeG did not block the spontaneous interaction of these inflammasome components in HEK293T cells, ruling out competitive inhibition of the NEK7–NLRP3 interaction. NEK7 is a key component that opens the closed cage-like structure of the NLRP3 oligomer and triggers ASC recruitment in response to mtROS [9,30]. Consistently, BeG inhibits mtROS production and the NEK7-NLRP3 interaction induced by R837. Although we initially hypothesized that BeG upregulates mitochondrial antioxidant genes (Txn2, Prdx3, Sod2), no significant changes in their expression were detected. Instead, transcriptome sequencing combined with functional validation revealed that BeG limits excessive mtROS production through an alternative mechanism—mitophagy. Mitophagy eliminates damaged mitochondria, thereby preventing the release of mtROS and mtDNA. In the absence of this elimination, these molecules would activate the NLRP3 inflammasome [20]. In addition, mitochondrial fusion and fission help preserve mitochondrial homeostasis and reduce excessive mtROS production. Tan et al. recently reported that maintaining the homeostasis of mitochondrial fusion–fission dynamics blocks the NEK7–NLRP3 interaction [40], which is consistent with the mechanistic observations in our study.

A previous study reported that BeG induces LC3-II accumulation [23], yet whether this reflects enhanced or blocked autophagic flux remained unclear. Using the mCherry-EGFP-LC3 reporter, we confirm that BeG increases autophagic flux. Notably, BeG promotes LC3–mitochondria colocalization without affecting LC3–NLRP3 association, further supporting selective mitophagy. Pathogens like Porphyromonas gingivalis impair mitophagy in BMDMs, driving IL-1β production [41]. Consistently, PINK1 knockout, which abrogates mitophagy, enhances alveolar osteoclast formation and bone resorption in a ligature-induced periodontitis model. These pathological effects are apparently dependent on NLRP3 activation [42,43]. Consistently, GO analysis in this study reveals that BeG concurrently upregulates mitophagy-related genes and downregulates osteoclast differentiation-related genes in inflamed BMDMs, underscoring a tight link between mitophagy and periodontal inflammation/osteoclast activity.

Furthermore, our results reveal that BeG promotes mitophagy by inhibiting the AKT–mTOR signaling axis [23]. This axis is known to negatively regulate both autophagy and mitophagy via phosphorylation of the ULK1–ATG13 complex [44]. Huang et al. recently demonstrated that BeG also inhibits the PI3K–AKT–mTOR pathway in chondrocytes and activates the PINK1/Parkin axis to promote mitophagy [45], suggesting that BeG exerts similar effects in multiple cell types. As a canonical negative regulator of mTOR, activated AMPK suppresses mTOR phosphorylation and enhances autophagic flux [46]. A prior study demonstrated that BeG enhances AMPK phosphorylation in HK-2 renal tubular epithelial cells [47]. Although such an effect was not observed in BMDMs or J774A.1 cells, this finding still contributes to understanding the mechanism by which BeG induces mitophagy in macrophages.

Kim et al. recently reported that BeG enhances the osteogenic differentiation capacity of human periodontal ligament cells [48], indicating that BeG not only functions as an anti-inflammatory agent but also promotes bone repair in inflammatory environments. These findings position BeG as a promising candidate for periodontitis therapy, although clinical translation remains challenging. BeG exhibits UVA-induced phototoxicity, a property clinically exploited in treating vitiligo and psoriasis [49]; however, the safety of non-topical routes has yet to be confirmed, and precautions against phototoxic effects are essential. Furthermore, BeG has poor oral bioavailability and is rapidly metabolized [22], and systemic administration may inhibit CYP3A4, a hepatic cytochrome P450 enzyme involved in drug metabolism [50]. Structural modification of BeG, combined with controlled-release platforms for local periodontal delivery (e.g., hydrogels or nanocarriers), represents a promising strategy to address these pharmacokinetic and safety limitations, enhance bioavailability, and achieve sustained, site-specific drug delivery to periodontal tissues.

Several limitations of this study should be noted. Although BeG primarily suppresses inflammasome activation by disrupting the NEK7–NLRP3 interaction, other mechanisms independent of mitophagy may also contribute to its effects. For example, BeG might induce post-translational modifications of NEK7 or other NLRP3 regulators, thereby preventing inflammasome assembly, or it could modulate upstream signaling pathways that control mitochondrial reactive oxygen species (mtROS) production. Mechanistically, the effect of MHY1485 in enhancing the NEK7–NLRP3 interaction via mTOR activation cannot be attributed solely to mitophagy inhibition, given its potential off-target effects. Moreover, mTOR is known to regulate GSDMD-NT pore formation [51], and the consequent potassium efflux through plasma membrane pores may further strengthen the NEK7–NLRP3 interaction [52]. Finally, since most findings were derived from in vitro cell systems and murine periodontitis models, the translational relevance to human disease and the clinical efficacy and safety of BeG remain to be established.

## 5. Conclusions

This study demonstrates that BeG effectively alleviates periodontal inflammation and alveolar bone loss in experimental periodontitis. Mechanistically, BeG blocks the NEK7–NLRP3 interaction and inflammasome activation by reducing mtROS levels, an effect potentially mediated by AKT–mTOR pathway inhibition that promotes mitophagy. These findings provide new insights into the anti-inflammatory effects of BeG and substantiate its therapeutic potential for periodontitis.

## Data Availability

The transcriptome sequencing data generated in this study have been deposited in the NCBI Sequence Read Archive (SRA) under BioProject accession number PRJNA1493853. All other relevant data are available within the manuscript and its Supplementary Materials. Further inquiries regarding data availability can be directed to the corresponding author.

## Supplementary Materials

The following supporting information can be downloaded at https://www.mdpi.com/article/10.3390/antiox15101304/s1, Figure S1: Effects of Bergapten on J774A.1 Cell Viability Assessed by CCK-8 Assay; Figure S2: Bergapten upregulates mRNA expression of mitophagy-related genes; Figure S3: Bergapten promotes mitochondrial LC3 recruitment and preserves mitochondrial membrane potential; Figure S4: Bergapten did not enhance autophagic clearance of the NLRP3 inflammasome nor induce AMPK phosphorylation; Table S1: Antibodies; Table S2: Sequences of RT-qPCR primers, Related to Figure 2; Table S3: Demographic information and clinical characteristics of periodontal healthy individuals and patients with periodontitis enrolled in this study. Original Western blot images.
