# Supplementary material for: Bergapten Alleviates Periodontitis by Suppressing NLRP3 Inflammasome Activation via Mitophagy-Induced Disruption of NEK7-NLRP3 Interaction
Source: Antioxidants (Basel). 2026 Oct 9;15(10):1304. doi: 10.3390/antiox15101304 (PMC13700881; doi:10.3390/antiox15101304)
Supplement: Supplementary file 1 [file antioxidants-15-01304-s001.zip › antioxidants-4430699-supplementary.pdf]

Supplementary Materials

Supplementary Information

S1. Supplementary Figures:

Figure S1. Effects of Bergapten on J774A.1 Cell Viability Assessed by CCK-8 Assay.

Figure S2. Bergapten upregulates mRNA expression of mitophagy-related genes.

Figure S3. Bergapten promotes mitochondrial LC3 recruitment and preserves mitochondrial membrane potential.

Figure S4. Bergapten did not enhance autophagic clearance of the NLRP3 inflammasome nor induce AMPK phosphorylation.

S2. Supplementary Tables

Table S1. Antibodies.

Table S2. Sequences of RT-qPCR primers, Related to Figure 2.

S3.Demographic information and clinical characteristics of periodontal healthy individuals and patients with periodontitis enrolled in this study

Table S3. Demographic information and clinical characteristics of periodontal healthy individuals and patients with periodontitis enrolled in this study.

S4.Original Western blot images

S1.Supplementary Figures

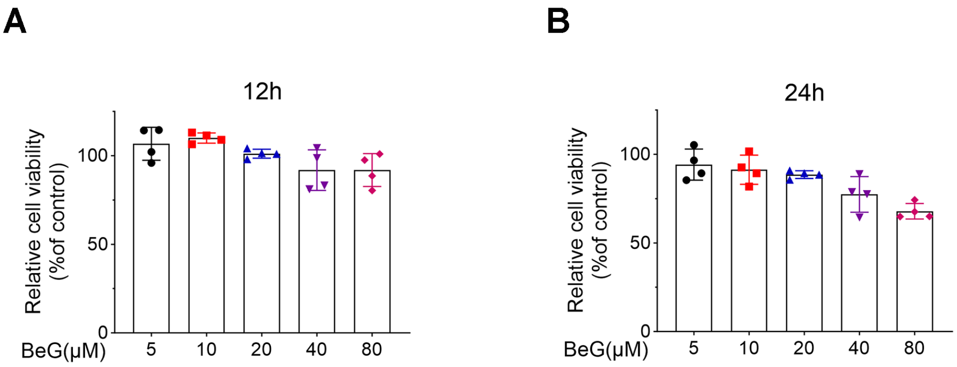

Figure S1. Effects of Bergapten on J774A.1 Cell Viability Assessed by CCK-8 Assay.

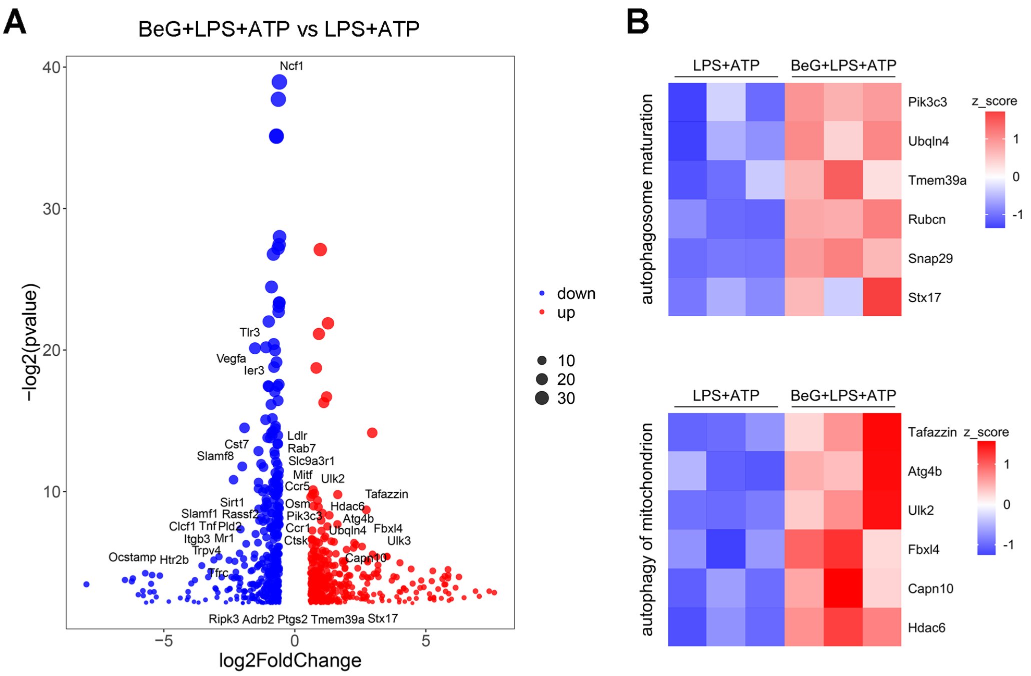

Figure S2. Bergapten upregulates mRNA expression of mitophagy-related genes. (A) Volcano plots illustrate the differentially expressed genes in LPS/ATP-stimulated BMDMs with or without BeG treatment. (B) The heatmap displays expression levels of genes associated with autophagosome maturation and mitophagy in LPS/ATP-stimulated BMDMs treated with or without BeG.

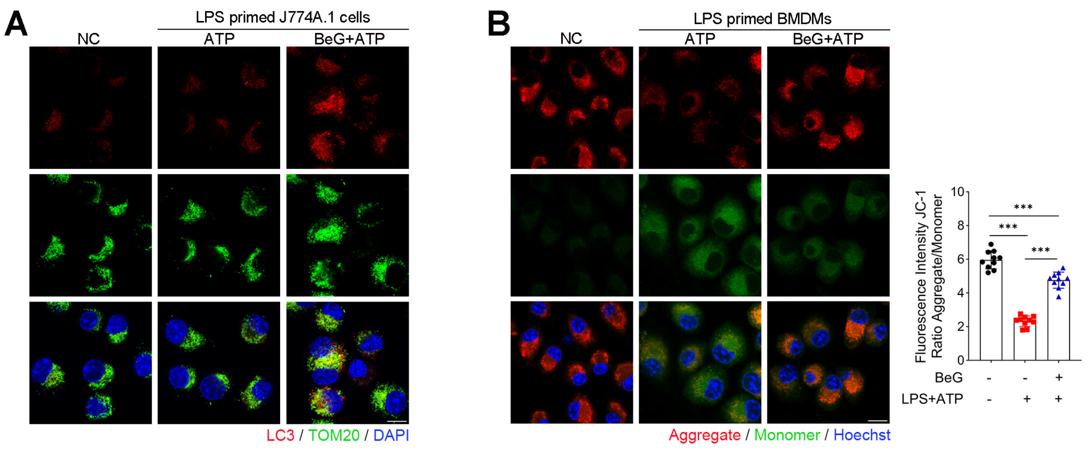

Figure S3. Bergapten enhances the recruitment of LC3 to mitochondria and maintains mitochondrial membrane potential. (A) Representative immunofluorescence images of LC3 and TOM20 staining in J774A.1 cells. Scale bar, 10 μm. (B) Mitochondrial membrane potential in BMDMs was assessed using JC-1 staining. Scale bar, 10 μm. Data are presented as mean ± SD. Statistical significance was indicated as follows: *P < 0.05, **P < 0.01, ***P < 0.001.

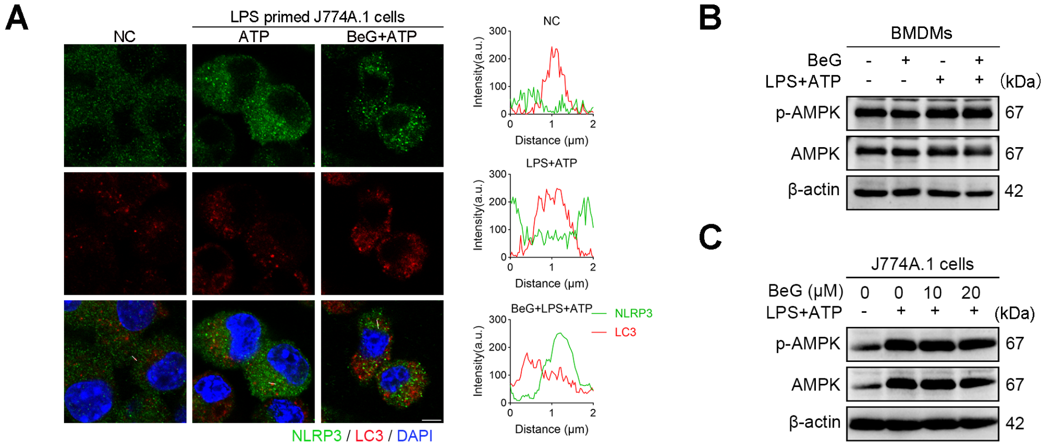

Figure S4. Bergapten did not enhance autophagic clearance of the NLRP3 inflammasome nor induce AMPK phosphorylation. (A) Representative immunofluorescence images of NLRP3 and LC3 staining in J774A.1 cells treated as indicated in the image. Scale bar, 5 μm. Fluorescence intensity profiles along the white lines are shown on the right. (B, C) BMDMs and J774A.1 cells were pretreated with

BeG prior to LPS/ATP stimulation. Protein levels of phosphorylated AMPK (p-AMPK) and total AMPK in whole-cell lysates were quantified by immunoblotting.

S2.Supplementary Tables

Table S1. Antibodies

Name	Source	Catalog Number	RRID
Mouse monoclonal anti-p-mTOR	Proteintech	Cat# 67778-1-Ig	RRID: 10029688
Rabbit polyclonal anti-mTOR	Proteintech	Cat# 28273-1-AP	N/A
Rabbit monoclonal anti-p-AMPK	Proteintech	Cat# 80209-6-RR	N/A
Mouse monoclonal anti-AMPK	Proteintech	Cat# 66536-1-Ig	N/A
Rabbit monoclonal anti-LC3B	Abmart	Cat# T55992	RRID: 10094157
Rabbit polyclonal anti-TOMM20	Proteintech	Cat# 11802 -1-AP	RRID: 00114739
Rabbit polyclonal anti-CD68	Bioss	Cat# bs-0649R	RRID: BC02037651
Mouse Monoclonal anti-NLRP3	Adipogen	Cat# AG-20B-0014	RRID: A43322207
Rabbit Monoclonal anti-GSDMD	HuaBio	Cat# HA721144	RRID: H660004008
Rabbit Monoclonal anti-p-AKT	HuaBio	Cat# ET1607-73	RRID: H650102058
Rabbit Monoclonal anti-AKT	HuaBio	Cat#HA721870	RRID: H640019023
Rabbit polyclonal anti-Cleaved-caspase1 (p20)	Wanleibio	Cat# WL02996a	RRID: N10272996
Rabbit polyclonal anti-IL-1B	Immunoway	Cat# YT5201	RRID: 3673A26ERK7
Mouse monoclonal anti-8-OHdG	Santa Cruz	Cat# sc-393871	RRID: H3122
Mouse monoclonal anti-ASC	Santa Cruz	Cat# sc-514414	RRID: J1922
Mouse monoclonal anti-NEK7	Santa Cruz	Cat# sc-393539	RRID: A3123
Mouse monoclonal anti-Myc-Tag	Abmart	Cat# M20002S	RRID: 10244366
Mouse monoclonal anti-HA-Tag	Abmart	Cat# M20003S	RRID: 10242961

Mouse monoclonal anti-His-Tag	Proteintech	Cat# 66005-1-Ig	RRID: 10052238
Rabbit polyclonal anti-β-actin	Abclonal	Cat# AC026	RRID: 3523062901
HRP Goat Anti-Rabbit IgG (H+L)	Boster	Cat# BA1054	N/A
HRP Goat Anti-Mouse IgG (H+L)	Boster	Cat# BA1051	N/A
Donkey Anti-Mouse IgG H&L (Alexa Fluor 488)	Abcam	Cat# ab150105	RRID: GR3462834-1
Donkey Anti-Rabbit IgG H&L (Alexa Fluor 647)	Abcam	Cat# ab150075	RRID: 1041692-38

Table S2. Sequences of RT-qPCR primers, Related to Figure 2.

Name	Species	Sequence of forward primer	Sequence of forward primer
D-loop	Mus musculus	AATCTACCATCCTCCGTGAAACC	TCAGTTTAGCTACCCCCAAGTTTAA
Tert	Mus musculus	CTAGCTCATGTGTCAAGACCCTCTT	GCCAGCACGTTTCTCTCGTT

S3.Demographic information and clinical characteristics of periodontal healthy individuals and patients with periodontitis enrolled in this study

Table S3. Demographic information and clinical characteristics of periodontal healthy individuals and patients with periodontitis enrolled in this study.

Characteristics	Periodontal healthy (n = 15)	Periodontitis (n = 15)
Gender (Male/Female)	6/9	8/7
Age (years, mean±SD)	33.2±8.53	46.53±9.51

Number of Tooth loss	0.00 ± 0.00	2.53±3.07
Smoking status (n, %)	0/0%	8/53.33%
Probing depth (mm)	2.4±0.49	6.37±0.94
Clinical attachment loss (mm)	0.2±0.31	7.4±0.69

S4.Original Western blot images

Figure 1C

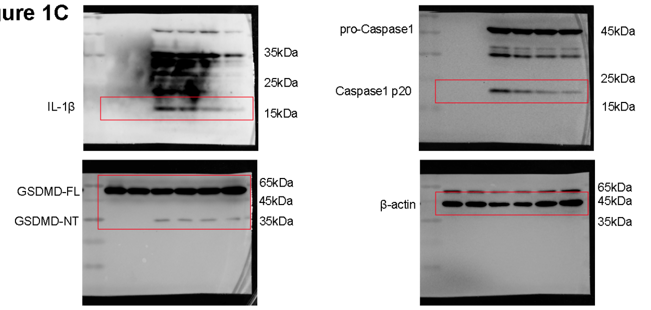

Figure 1D

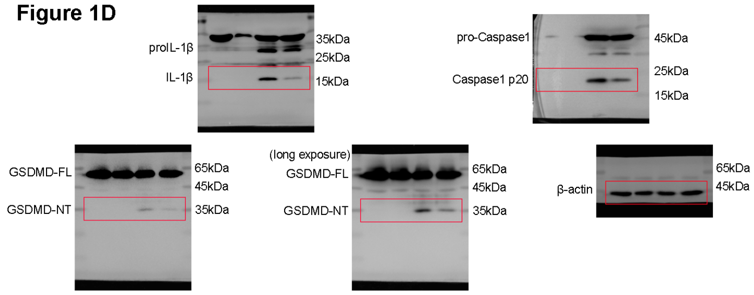

Figure 2I

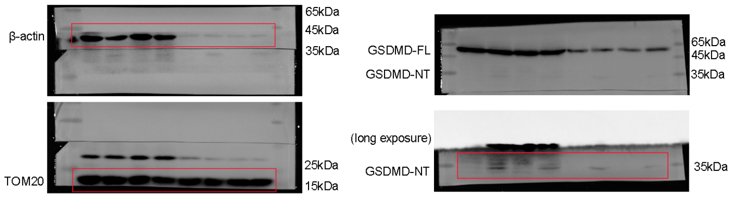

Figure 3A

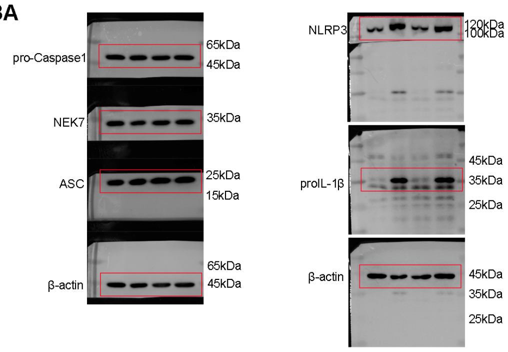

Figure 3B

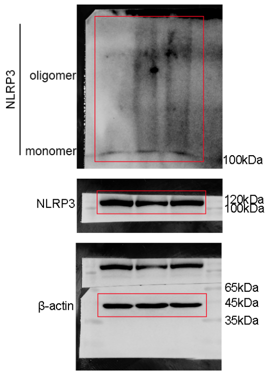

Figure 3C

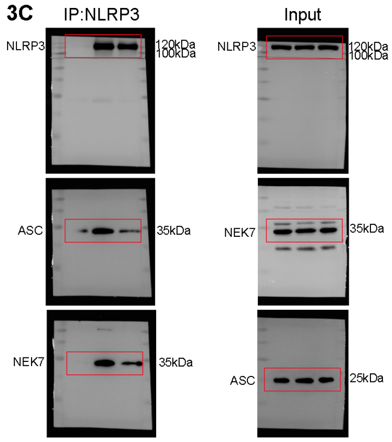

Figure 4E

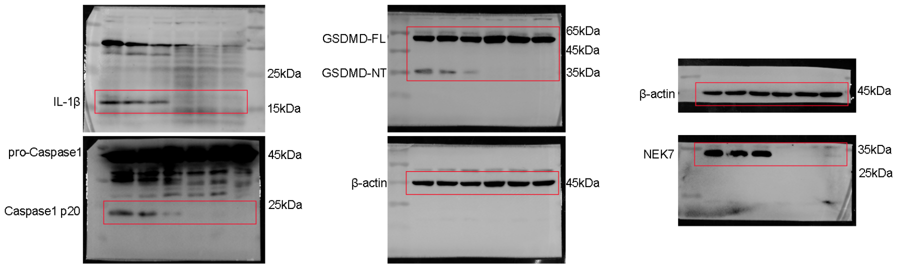

Figure 5F

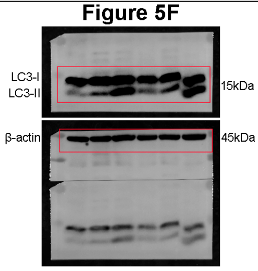

Figure 5G

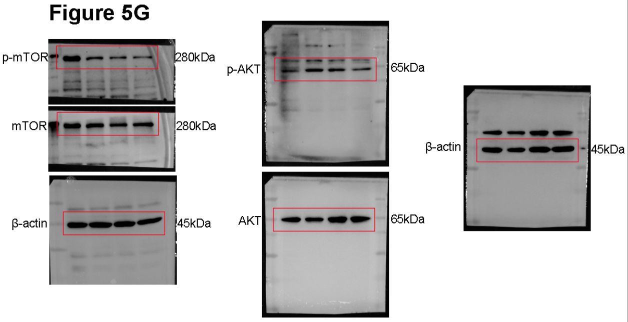

Figure 6G

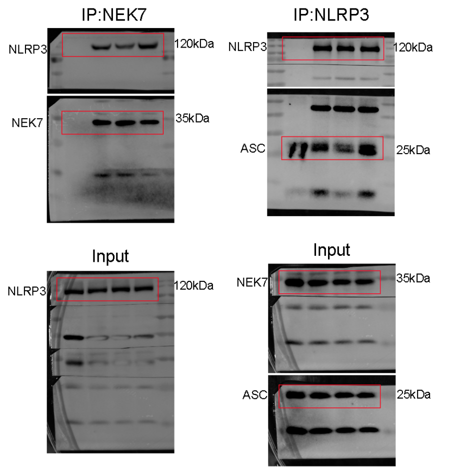

Figure 6H

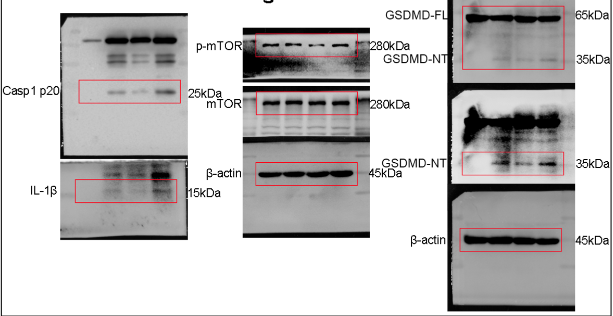

Figure S3B

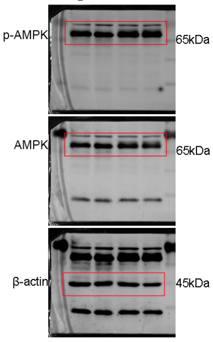

Figure S3C

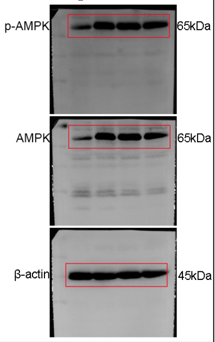

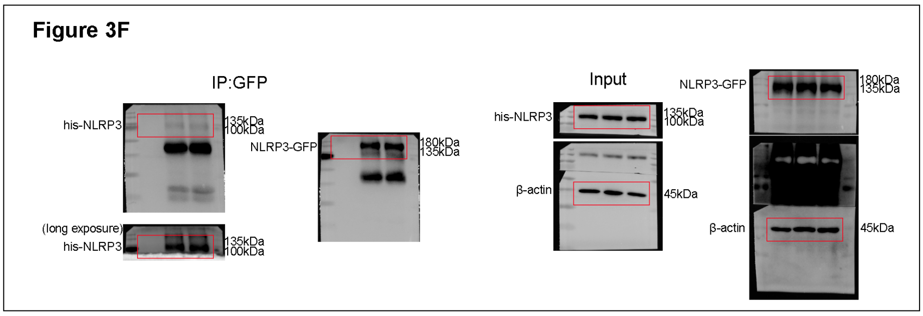
Figure 3F
IP:GFP
his-NLRP3
135kDa
100kDa
NLRP3-GFP
180kDa
135kDa
(long exposure)
his-NLRP3
135kDa
100kDa
Input
his-NLRP3
135kDa
100kDa
β-actin
45kDa
NLRP3-GFP
180kDa
135kDa
β-actin
45kDa

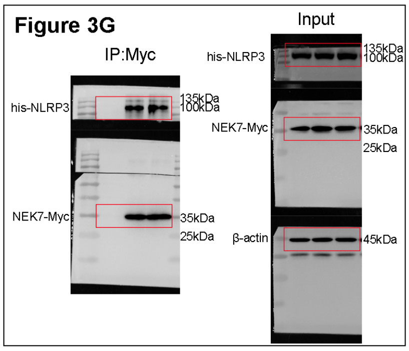
Figure 3G
IP:Myc
his-NLRP3
135kDa
100kDa
NEK7-Myc
35kDa
25kDa
Input
his-NLRP3
135kDa
100kDa
NEK7-Myc
35kDa
25kDa
β-actin
45kDa

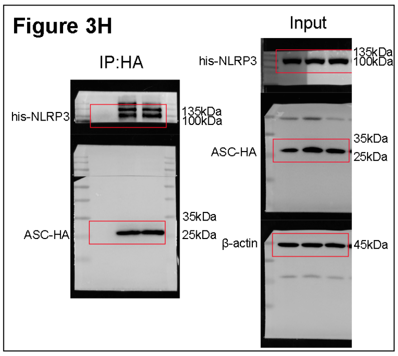
Figure 3H
IP:HA
his-NLRP3
135kDa
100kDa
ASC-HA
35kDa
25kDa
Input
his-NLRP3
135kDa
100kDa
ASC-HA
35kDa
25kDa
β-actin
45kDa

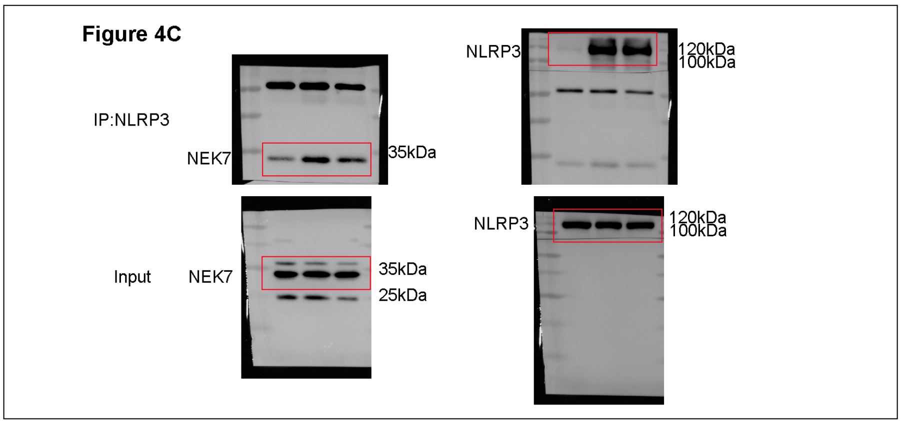
Figure 4C
IP:NLRP3
NEK7
35kDa
NLRP3
120kDa
100kDa
Input
NEK7
35kDa
25kDa
NLRP3
120kDa
100kDa

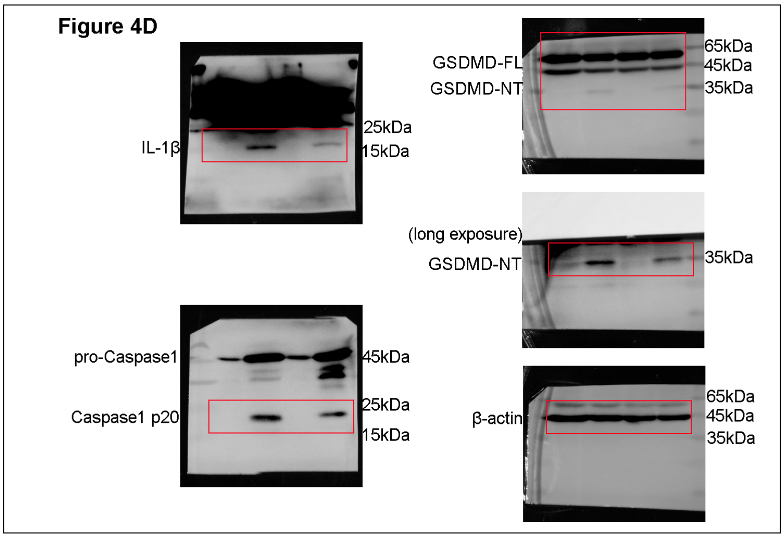
Figure 4D
IL-1β
25kDa
15kDa
GSDMD-FL
GSDMD-NT
65kDa
45kDa
35kDa
(long exposure)
GSDMD-NT
35kDa
pro-Caspase1
Caspase1 p20
45kDa
25kDa
15kDa
β-actin
65kDa
45kDa
35kDa
